# Health insurance coverage and poverty status of postpartum women in the United States in 2019: an ACS-PUMS population-based cross-sectional study

**DOI:** 10.1186/s12889-023-17087-4

**Published:** 2023-11-08

**Authors:** Bojung Seo, Jack Edward Turman, Hongmei Nan

**Affiliations:** 1 Department of Epidemiology, Richard M. Fairbanks School of Public Health, Indiana University, Indianapolis, IN USA; 2 Department of Social and Behavioral Sciences, Richard M. Fairbanks School of Public Health, Indiana University, 1050 Wishard Blvd, Room 6042, 46202 Indianapolis, IN USA; 3 Department of Pediatrics, School of Medicine, Indiana University, Indianapolis, IN USA; 4 Department of Global Health, Richard M. Fairbanks School of Public Health, Indiana University, 1050 Wishard Blvd, 6110, 46202 Indianapolis, RG, IN USA

**Keywords:** Postpartum period, Poverty, Universal Health Insurance, Health Disparate, Minority and vulnerable populations, Health Policy

## Abstract

**Background:**

A quarter of United States (US) postpartum women still report unmet health care needs and health care unaffordability. We aimed to study associations between receipt of health insurance coverage and poverty status/receipt of government financial support and determine coverage gaps overall and by social factors among US postpartum women in poverty.

**Methods:**

This study design is a cross-sectional study using secondary data. We included women who gave birth within the last 12 months from 2019 American Community Survey Public Use Microdata Sample. Poverty was defined as having an income-to-poverty ratio of less than 100%. We explored Medicaid/government medical assistance gaps among women in poverty. To examine the associations between Medicaid/government medical assistance (exposures) and poverty/government financial support (outcomes), we used age-, race-, and multivariable-adjusted logistic regression models. We also evaluated the associations of state, race, citizenship status, or language other than English spoken at home (exposures) with receipt of Medicaid/government medical assistance (outcomes) among women in poverty through multivariable-adjusted logistic regression.

**Results:**

It was notable that 35.6% of US postpartum women in poverty did not have Medicaid/government medical assistance and only a small proportion received public assistance income (9.8%)/supplementary security income (3.1%). Women with Medicaid/government medical assistance, compared with those without the coverage, had statistically significantly higher odds of poverty [adjusted odds ratio (aOR): 3.15, 95% confidence interval (95% CI): 2.85–3.48], having public assistance income (aOR: 24.52 [95% CI: 17.31–34.73]), or having supplementary security income (aOR: 4.22 [95% CI: 2.81–6.36]). Also, among postpartum women in poverty, women in states that had not expanded Medicaid, those of Asian or other race, non-US citizens, and those speaking another language had statistically significantly higher odds of not receiving Medicaid/government medical assistance [aORs (95% CIs): 2.93 (2.55–3.37); 1.30 (1.04–1.63); 3.65 (3.05–4.38); and 2.08 (1.86–2.32), respectively].

**Conclusions:**

Our results showed that the receipt of Medicaid/government medical assistance is significantly associated with poverty and having government financial support. However, postpartum women in poverty still had Medicaid/government medical assistance gaps, especially those who lived in states that had not expanded Medicaid, those of Asian or other races, non-US citizens, and other language speakers.

**Supplementary Information:**

The online version contains supplementary material available at 10.1186/s12889-023-17087-4.

## Background

The postpartum period, defined as up to 1 year after delivery [1], is of particular importance in maternal and child health. The Office of Disease Prevention and Health Promotion highlights the importance of postpartum preventive intervention or screening programs [2]. Specifically, such screening in postpartum women may help mothers to receive early diagnosis and management of pregnancy complications, such as hypertensive disorders of pregnancy or gestational diabetes, which may lead to reduction in risk of long-term cardiovascular diseases [3]. Ensuring health care for the poorest mothers and infants has been a policy goal in the United States (US) for decades [4]. To improve coverage and affordability of recommended health care services for postpartum women, health care policies were enacted and expanded, e.g., enactment of Medicaid in 1965, the great expansion of eligibility for its coverage of the expenses of postpartum women in the 1980s, the Pregnancy Discrimination Act of 1978 (Pub. L. 95–555) [4], and the Patient Protection and Affordable Care Act (sometimes known as ACA, PPACA, or ‘Obamacare’) in 2010. These policies with subsidies benefited all reproductive-aged women living on low incomes by improving insurance coverage and reducing cost-related barriers to care [5, 6]. However, many women with low incomes still experienced unmet needs and transitions in health care insurance during the postpartum period [7, 8]. Between 2013 and 2018, approximately a quarter of US postpartum women reported financial hardship, and postpartum women with lower incomes were less likely to afford health care than those with higher incomes [9]. Such insufficient medical access played a partial role in negative infant [10, 11] and maternal health outcomes [12–14]. In fact, the US lagged behind other high-income countries in terms of maternal and infant health. In 2018, there were 17.4 maternal deaths for every 100,000 live births in the US, a ratio more than double that of most other rich nations [15], and in 2019, the US had the fifth worst infant mortality rate (5.8 deaths per 1,000 live births) among 36 OECD (Organisation for Economic Co-operation and Development) countries, whose average rate is 3.8 [16].

Such high maternal and infant mortality rates raised several questions. How well were current policies for postpartum women in poverty operated? Did women with public health coverage receive government financial support as well? Thus, we sought to study whether postpartum women in poverty have benefited from current health insurance coverage and received government financial support, and to further study coverage gaps in socially vulnerable groups in poverty, such as non-US citizens. We hypothesized that if governmental health policies are well implemented for postpartum women in poverty, the associations between receipt of public health insurance and poverty/government financial support would be significant. We also hypothesized that socially vulnerable postpartum women in poverty would have larger public health coverage gaps. Therefore, we examined the associations between health insurance coverage and poverty/government financial support. We also comprehensively studied the coverage gaps among postpartum women in poverty as well as whether significant differences in public health coverage gaps existed among the poverty group stratified by race, citizenship status, language in use, and state (by Medicaid expansion status).

## Methods

### Study data and population

In this cross-sectional study, we used secondary data from the 2019 Public Use Microdata Sample (PUMS), a sample of full American Community Survey (ACS) microdata, which contains demographic and socioeconomic features including incomes and health insurance and health coverages plans of individuals corresponding to approximately 1% of the US population [17]. Response rates of the 2019 ACS were very high at 86.0% for housing unit and at 90.9% for group quarters/persons [18]. Furthermore, ACS PUMS data are of very high-quality, given measures to control biases or errors [19]. In addition, to control measurement and processing error, ACS carefully monitored the work of interviewers, prepared field staff for their tasks, reinterviewed a sample of the households interviewed by computer-assisted personal interviewers, and imputed any remaining incomplete or inconsistent information in the collected data during the final content-editing phase [19]. To check if our initial study dataset was correctly downloaded and set up before conducting specific analyses, we compared our weighted frequencies and corresponding standard error (SE) with PUMS estimates for User Verification officially provided by the US Census Bureau together with the comparison of record counts (https://www.census.gov/programs-surveys/acs/microdata/documentation.2019.html). The US data include Washington, DC and all states, but excludes Puerto Rico [17]. We restricted our initial dataset to self-identified women who have given birth to any children in the last 12 months. The study data were collected every day of the year 2019 [20] and analyzed between December, 2021 and May, 2022. Because most ACS PUMS responses were modified to protect confidentiality of the survey respondents, the publicly available, deidentified data used in this study was considered an exemption by the institutional review board of Indiana University and informed consent did not need to be obtained. This study followed the Strengthening the Reporting of Observational Studies in Epidemiology (STROBE) reporting guidelines.

### Health insurance or health coverage plans

In this study, health insurance or health coverage plans encompassed: (1) Medicaid or medical assistance or any kind of government assistance for those with low incomes or a disability (hereinafter referred to as Medicaid or any kind of government medical assistance), (2) private health insurance, and (3) insurance through a current or former employer or union of the interviewee or a family member of the interviewee (hereinafter referred to as insurance through an employer/union). Medicaid or any kind of government medical assistance was considered public health coverage as a primary exposure in the analyses of this study. Private health insurance was defined by integrating the following health insurance or coverage plan types: insurance through an employer/union, insurance purchased directly from an insurance company by the respondent or another family member, and TRICARE or other military health care [21]. Insurance through an employer/union was also individually examined in the analyses, separately from private health insurance.

### Poverty status and other poverty-related outcomes

All questions on our outcomes specified a period covering the last 12 months [21]. Because ACS defined the income-to-poverty ratio as the Federal Poverty Level (FPL), we used income-to-poverty ratio to define poverty status. Based on a poverty definition of the US Census Bureau, we defined the poverty group as women with income-to-poverty ratio of less than 100%, and the non-poverty group as women with income-to-poverty ratio of greater than or equal to 100% [22]. We also included continuous variables of income-to-poverty ratio, and total personal income, a sum added altogether with total amounts of various types of incomes. The total personal income values were converted to 2019 constant dollars to reflect inflation by using consumer price index [23]; The total personal income values were further adjusted to reflect state-level cost of living differentials using the average cost of living index by state [24]. Other poverty-related outcomes were whether a postpartum woman had government financial support (i.e., public assistance income or supplemental security income). Specifically, public assistance income used interchangeably with cash public assistance included general assistance and Temporary Assistance to Needy Families and excludes separate payments received for hospital or other medical care (vendor payments) [21]. This did not include supplemental security income or noncash benefits such as food stamps [21]. Supplemental security income was a nationwide US assistance program administered by the Social Security Administration that guarantees a minimum level of income for needy aged, blind, or disabled individuals [21]. Poverty threshold-related information, survey questions for health insurance plans and incomes, and more detailed explanations on our outcomes were described in the **Supplementary Material**.

### Social factors and state

We chose race, citizenship status, and language other than English spoken at home as social factors of interest for examination of populations more vulnerable to uninsurance among postpartum women in poverty. For this examination, we classified citizenship status as non-US citizens vs. US citizens and race as Black/African American, American Indian/Alaska Native/Native Hawaiian/Other Pacific Islander, Asian/some other race, two or more races vs. White; we were not able to further examine race with more detailed categories (e.g., non-Hispanic White, non-Hispanic Black etc.) due to the limited sample size. Non-US citizens were defined as non-citizens of the US or women born abroad of American parents, and US citizens were defined as US citizens by naturalization or women born in the US, in Puerto Rico, Guam, the US Virgin Islands, or the Northern Marianas. We further included a variable of “state” to determine whether Medicaid expansion is related to the larger coverage gaps. We categorized states (unexpanded vs. expanded) based on the states’ decisions about adopting the Medicaid expansion as of January 1, 2019 [25]. The detailed list of Medicaid expansion states is presented in the Supplementary Material.

### Statistical analysis

The ACS PUMS was a complex sample design with its weighting variables to represent the actual population [17]. We used PUMS weighting variables in all analyses to calculate not only weighted estimates, but also accurate measures of uncertainty of the weighted estimates (i.e., SEs). Specifically, we used the person’s weight (PWGTP) for generation of the statistics on individuals, and 80 replicate weights (PWGTP1 to PWGTP80) for estimation of the SEs. To obtain the SE, we used a formula of successive difference replication SE [17]: $\sqrt{VAR \left(x\right)}=\sqrt{\frac{4}{80}\sum _{r=1}^{80}{({x}_{r}-x)}^{2}}$ (${x}_{r}$ is a r^th^ replicate estimate, and $x$ is the full PUMS weighted estimate).

Continuous variables were presented as weighted mean (SE of mean) and categorical variables were presented as weighted frequency (weighted percentage) and SE of weighted frequency. We also compared survey-weighted proportions or means of demographic and socioeconomic characteristics, considered as potential confounding factors, between poverty and non-poverty groups using Rao-Scott chi-square tests or t-tests, from which we singled out the final covariates related to poverty status. In terms of nativity, even though it was not statistically significant in the Rao-Scott chi-square test, we also included it for adjustment based on the literature [26, 27]. Such selected covariates were used in our multivariable-adjusted logistic and linear regression models for adjustment.

To answer the questions – *How well are current policies for postpartum women in poverty operated? Do women with public health coverage receive government financial support as well?* – we examined the associations between Medicaid or any kind of government medical assistance (i.e., exposures – reference categories: no receipt of Medicaid or any kind of government medical assistance) and poverty status/government financial support (i.e., outcomes – reference categories: no poverty/no receipt of government financial support). For examination of each respective association, we utilized age- and race-adjusted logistic regression models as well as multivariable-adjusted logistic regression models, yielding adjusted odds ratios (aORs) and corresponding 95% confidence intervals (95% CIs). In the multivariable-adjusted logistic regression models, in addition to age and race, we adjusted for potential confounding factors, including region, nativity, marital status, educational attainment, language other than English spoken at home, ambulatory difficulty, cognitive difficulty, disability, and employment status. Additionally, we also estimated relative risks of Medicaid or any kind of government medical assistance (Yes vs. No).

In order to answer the question – *Which subgroups are most vulnerable to uninsurance among postpartum women in poverty?* – we conducted stratified analyses according to state (Medicaid non-expanded states vs. expanded states), race (Black/African American, American Indian/Alaska Native/Native Hawaiian/Other Pacific Islander, Asian/some other race, and two or more races vs. White), citizenship status (non-US citizens vs. US citizens), and language used at home (other language vs. English only). We also explored whether associations between each such factor (i.e., exposures - reference categories: expanded states; White or Black/African American; US citizens; English only) and receipt of Medicaid or any kind of government medical assistance (i.e., outcomes - reference categories: having Medicaid or any kind of government medical assistance) are statistically significant and how strong they are by using multivariable-adjusted logistic regression models. Also, we calculated relative risks of state (Medicaid non-expanded states vs. expanded states), race (Black/African American, American Indian/Alaska Native/Native Hawaiian/Other Pacific Islander, Asian/some other race, and two or more races vs. White), citizenship status (non-US citizens vs. US citizens), and language used at home (other language vs. English only). Additionally, we analyzed each state’s Medicaid or any kind of government medical assistance gaps among those in poverty to acquire state-by-state coverage gaps in conjunction with each state’s Medicaid expansion status.

Lastly, as supplementary analyses, we also examined the relationships between health insurance coverage (exposure) and each continuous variable of poverty status, i.e., total person’s income and income-to-poverty ratio (outcomes); we conducted age- and race-adjusted and multivariable-adjusted linear regression, yielding adjusted coefficient (a*β*) and corresponding 95% CI.

All analyses were conducted using SURVEY procedures, provided for complex survey data, in SAS software (Unix 9.4; SAS Institute, Inc., Cary, North Carolina), and statistical significance was set at 2-tailed *P* values < 0.05. To adjust the *P* values from multiple testing for the same outcome variable, we applied the conservative Bonferroni correction method, which multiplied the raw *P* values by the number of tests [28].

## Results

The study cohort included a total of 34,257 postpartum women, weighted to represent 3,839,270 women. 20.9% (802,594 postpartum women) lived in poverty during the postpartum period. The demographic and socioeconomic characteristics according to poverty status of the study population in the US are presented in Table 1. Black or African American women made up 24.8% of women in poverty but only 11.9% of women without poverty; White people represented 57.7% of women in poverty and 70.4% of women without poverty. Compared to women without poverty, women in poverty were younger [mean (SE): 28.5 (0.1) vs. 31.1 (0.04); range: 15–50 in both groups], more likely to be never married (59.2% vs. 20.1%), unemployed (9.4% vs. 2.7%), and less educated (≥ Associate degree or Bachelor degree: 13.2% vs. 52.5%). Additionally, among the poverty group, only a small proportion had public assistance income (9.8%) or supplementary security income (3.1%).

**Table 1:** Comparisons of demographic and socioeconomic characteristics according to poverty status using Rao-Scott chi-square tests or t-tests ^f^

	Poverty		No poverty	
Characteristics	N (%) ^a^	SE ^b^	N (%) ^a^	SE ^b^
**Total Women**	802,594 (20.9)	13,560	3,036,676 (79.1)	21,612
**Age** ^**c**^ **(years)**	28.5 (0.1)		31.1 (0.04)	
**Race**				
White	463,466 (57.7)	10,575	2,136,986 (70.4)	19,328
Black or African American	198,672 (24.8)	6899	362,135 (11.9)	8742
American Indian, Alaska Native, or Native Hawaiian and Other Pacific Islander	16,744 (2.1)	1650	27,510 (0.9)	2210
Asian or some other race	91,659 (11.4)	4609	409,849 (13.5)	9683
Two or more races	32,053 (4.0)	2529	100,196 (3.3)	4469
**Region**				
Northeast	114,444 (14.3)	4806	480,572 (15.8)	9347
Midwest	168,928 (21.0)	5719	649,350 (21.4)	10,651
South	353,900 (44.1)	9191	1,146,184 (37.7)	12,404
West	165,322 (20.6)	5805	760,570 (25.0)	9466
**Nativity**				
Native	643,252 (80.1)	12,484	2,461,811 (81.1)	20,687
Foreign born	159,342 (19.9)	5398	574,865 (18.9)	11,390
**Citizenship status**				
Born in the US	628,554 (78.3)	12,106	2,405,622 (79.2)	19,625
Born in Puerto Rico, Guam, the US Virgin Islands, or the Northern Marianas	9418 (1.2)	1471	17,919 (0.6)	1924
Born abroad of American parent(s)	5280 (0.7)	936	38,270 (1.3)	3224
US citizen by naturalization	34,952 (4.4)	2363	217,472 (7.2)	6228
Not a citizen of the US	124,390 (15.5)	4856	357,393 (11.8)	9222
**Marital status**				
Now married, spouse present	181,177 (22.6)	6400	2,176,481 (71.7)	20,266
Now married, spouse absent	41,996 (5.2)	3026	112,065 (3.7)	5574
Widowed	5603 (0.7)	1077	7307 (0.2)	1053
Divorced	61,996 (7.7)	3244	91,268 (3.0)	4138
Separated	36,804 (4.6)	2623	40,252 (1.3)	3031
Never married	475,018 (59.2)	10,527	609,303 (20.1)	10,253
**Educational attainment**				
Less than high school	191,452 (23.9)	6592	223,135 (7.3)	6800
Regular high school diploma	254,041 (31.7)	7751	519,103 (17.1)	9814
GED or alternative credential	42,648 (5.3)	2794	69,662 (2.3)	3300
Some college, but no degree	207,914 (25.9)	6321	629,642 (20.7)	11,810
Associate degree or Bachelor’s degree	95,653 (11.9)	4178	1,078,919 (35.5)	14,462
Master’s degree	8379 (1.0)	1248	383,487 (12.6)	7063
Doctorate degree or Professional degree beyond a Bachelor’s degree	2507 (0.3)	594	132,728 (4.4)	4493
**Language other than English spoken at home**				
Yes, speaks another language	254,999 (31.8)	7912	858,681 (28.3)	12,749
**Ambulatory difficulty**, Yes	17,237 (2.1)	1605	23,229 (0.8)	2007
**Cognitive difficulty**, Yes	36,788 (4.6)	2604	51,806 (1.7)	2931
**Disability**, With a disability	69,872 (8.7)	3974	113,085 (3.7)	4385
**Employment status**				
Civilian employed, at work	240,554 (30.0)	8170	1,845,013 (60.8)	17,232
Civilian employed, with a job but not at work	38,031 (4.7)	2776	237,998 (7.8)	7438
Unemployed	75,135 (9.4)	4197	81,057 (2.7)	3537
Armed forces, at work	330 (0.04)	188	10,766 (0.4)	1317
Not in labor force	447,108 (55.8)	9408	858,078 (28.3)	11,173
Armed forces, with a job but not at work			135 (0.005)	115
**Income** ^**d**^				
**Total person’s income** ^**c, e**^ **(dollars)**	5388.9 (118.6)		33592.0 (301.3)	
**Income-to-poverty ratio** ^**c**^ **(%)**	43.0 (1.0)		333.0 (1.0)	
**Public assistance income**, Yes	78,816 (9.8)	4168	66,901 (2.2)	3996
**Supplementary security income**, Yes	25,249 (3.1)	2185	23,170 (0.8)	2020

Abbreviation: N, number; SE, standard errors; GED, general educational development

Note: Poverty (< 100%) vs. no poverty (≥ 100%) was defined according to income-to-poverty ratio.

^a^ Weighted frequency (weighted percentage); ^b^ Standard errors of weighted frequency; ^c^ Weighted mean (standard errors of mean); ^d^ Income variables were adjusted into 2019 constant dollars using inflation adjustment factor; ^e^ We adjusted the total personal income with the average cost of living index by state obtained from https://www.insure.com/cost-of-living-by-state.html; ^f^ All *P* values were < 0.0001, except for Nativity (*P* = 0.2147).

We examined the coverage gaps among women in poverty as well as the associations between Medicaid or any kind of government medical assistance and poverty status/government financial support (Table 2). 35.6% did not receive Medicaid or any kind of government medical assistance. Postpartum women with Medicaid or any kind of government medical assistance had statistically significantly higher odds of poverty compared to those without coverage (aOR, 95% CI: 3.15, 2.85 ~ 3.48). Among those with Medicaid or any kind of government medical assistance, 87.7% and 96.3% did not receive public assistance income and supplementary security income, respectively. Those with Medicaid or any kind of government medical assistance had statistically significantly higher odds of having public assistance income and supplementary security income (aORs, 95% CIs: 23.37, 16.49 ~ 33.12; 4.22, 2.81 ~ 6.36), compared with women who had neither. Relative risks of Medicaid or any kind of government medical assistance in multivariable adjusted models were also all statistically significant for each of outcomes (poverty, having public assistance income, and having supplementary security income) (Table 2).

**Table 2:** Associations between Medicaid or any kind of government medical assistance and poverty/government financial support using logistic regression

	Poverty ^a^		Having public assistance income ^b^		Having supplementary security income ^b^	
	No	Yes	No	Yes	No	Yes
Medicaid, medical assistance, or any kind of government-assistance						
Yes, n (%)	576,529 (19.0)	516,525 (64.4)	958,419 (87.7)	134,635 (12.3)	1,052,931 (96.3)	40,123 (3.7)
No, n (%)	2,460,147 (81.0)	286,069 (35.6)	2,735,134 (99.6)	11,082 (0.4)	2,737,920 (99.7)	8296 (0.3)
Age and race adjusted model (Ref.: no) ^c^						
* Odds ratios*	Ref.	6.62 (6.09 ~ 7.19)	Ref.	34.00 (25.58 ~ 45.20)	Ref.	15.12 (10.85 ~ 21.07)
* Relative risks*	Ref.	3.99 (3.74 ~ 4.24)	Ref.	29.74 (21.59 ~ 37.90)	Ref.	14.56 (9.84 ~ 19.28)
Multivariable adjusted model (Ref.: no) ^c^						
* Odds ratios*	Ref.	3.15 (2.85 ~ 3.48)	Ref.	24.52 (17.31 ~ 34.73)	Ref.	4.22 (2.81 ~ 6.36)
* Relative risks*	Ref.	2.88 (2.44 ~ 3.32)	Ref.	20.68 (13.53 ~ 27.84)	Ref.	4.07 (2.50 ~ 5.65)

Note: Poverty (< 100%) vs. no poverty (≥ 100%) was defined according to income-to-poverty ratio. In this table, public health coverage is the exposure and poverty status/government financial support is the outcome of interest

^a^ In multivariable adjusted logistic models, we adjusted age (continuous), race (White alone, Black or African American alone, American Indian, Alaska Native, or Native Hawaiian and Other Pacific Islander, Asian or some other race, two or more races), region (northeast, midwest, south, west), nativity (native, foreign born), marital status (now married spouse present, now married spouse absent, widowed, divorced, separated, never married), educational attainment (less than high school, regular high school diploma, GED or alternative credential, some college but no degree, associate degree or bachelor’s degree, master’s degree, doctorate degree or professional degree beyond a bachelor’s degree), language other than English spoken at home (yes speaks another language, no speaks only English), ambulatory difficulty (yes, no), cognitive difficulty (yes, no), disability (with a disability, without a disability), employment status (civilian employed at work, civilian employed with a job but not at work, unemployed, armed forces at work, not in labor force, armed forces with a job but not at work); ^b^ In multivariable adjusted logistic models, we excluded region and employment status and added class of worker (employee of a private for-profit company or business, or of an individual, for wages, salary, or commissions, employee of a private not-for-profit, tax-exempt, or charitable organization, local government employee, state government employee, federal government employee, self-employed in own not incorporated business professional practice or farm, self-employed in own incorporated business professional practice or farm, working without pay in family business or farm, unemployed and last worked 5 years ago or earlier or never worked) for covariate adjustment; ^c^ Values indicate adjusted odds ratios/relative risks (95% confidence intervals)

In Table 3, we explore the coverage gaps in the poverty group stratified by state, race, citizenship status, and language spoken at home as well as associations between state/race/citizenship/language and receipt of Medicaid or any kind of government medical assistance. 48.6% of women who lived in Medicaid-unexpanded states, 47.6% of Asian or some other race, 64.2% of non-US citizens, and 50.1% of other language speakers did not have Medicaid or any kind of government medical assistance. In all multivariable-adjusted logistic regression models, *P* values were < 0.0004 after conservative Bonferroni correction. Women in unexpanded states, of Asian or some other race, non-US citizens, and those speaking another language had statistically significantly higher odds of not receiving Medicaid or any kind of government medical assistance (aORs, 95% CIs: 2.93, 2.55 ~ 3.37; 1.30, 1.04 ~ 1.63; 3.65, 3.05 ~ 4.38; 2.08, 1.86 ~ 2.32). Similar coverage gaps and association patterns were observed in the analyses for Medicaid or any other kind of government medical assistance. Relative risks of state, race, citizen status, and language other than English spoken at home were not statistically significant for Medicaid or any kind of government medical assistance (Table 3).

**Table 3:** Associations between state/race/citizenship/language and Medicaid or any kind of government medical assistance among postpartum women in poverty using logistic regression ^c^

		Medicaid, medical assistance, or any kind of government-assistance				
		Yes	No			
**State by status of Medicaid expansion** ^d^						
Not expanded, n (%)		179,524 (51.4)	169,427 (48.6)			
Expanded, n (%)		337,001 (74.3)	116,642 (25.7)			
Multivariable adjusted model (Ref.: expanded) ^a, b^					*Odds ratios*	*Relative risks*
		Ref.	2.93 (2.55 ~ 3.37)			1.18 (0.91 ~ 1.44)
**Race**						
Black or African American, n (%)		146,757 (73.9)	51,915 (26.1)			
American Indian, Alaska Native, or Native Hawaiian and Other Pacific Islander, n (%)		12,092 (72.2)	4652 (27.8)			
Asian or some other race, n (%)		48,060 (52.4)	43,599 (47.6)			
Two or more races, n (%)		21,304 (66.5)	10,749 (33.5)			
White, n (%)		288,312 (62.2)	175,154 (37.8)			
Multivariable adjusted model (Ref.: White) ^a, b^			*Odds ratios*			*Relative risks*
Black or African American		Ref.	0.60 (0.50 ~ 0.73)			0.92 (0.81 ~ 1.03)
American Indian, Alaska Native, or Native Hawaiian and Other Pacific Islander		Ref.	0.68 (0.44 ~ 1.08)			0.94 (0.83 ~ 1.05)
Asian or some other race		Ref.	1.30 (1.04 ~ 1.63)			1.03 (0.98 ~ 1.09)
Two or More races		Ref.	0.94 (0.64 ~ 1.38)			0.99 (0.94 ~ 1.05)
**Citizenship status** ^e^						
Non-US citizens, n (%)		46,451 (35.8)	83,219 (64.2)			
US citizens, n (%)		470,074 (69.9)	202,850 (30.1)			
Multivariable adjusted model (Ref.: US citizens) ^a, b^			*Odds ratios*			*Relative risks*
		Ref.		3.65 (3.05 ~ 4.38)		1.14 (0.93 ~ 1.36)
**Language other than English spoken at home**						
Yes, speaks another language, n (%)		127,362 (49.9)	127,637 (50.1)			
No, speaks only English, n (%)		389,163 (71.1)	158,432 (28.9)			
Multivariable adjusted model (Ref.: no) ^a, b^			*Odds ratios*			*Relative risks*
		Ref.	2.08 (1.86 ~ 2.32)			1.10 (0.96 ~ 1.24)

Note: Poverty (< 100%) vs. no poverty (≥ 100%) was defined according to income-to-poverty ratio. In this table, state/race/citizenship status/language in use at home are exposures and public health coverage/Medicaid, medical assistance, or any kind of government-assistance are outcomes of interest

^a^ In all multivariable adjusted logistic models, we adjusted age (continuous), marital status (now married spouse present, now married spouse absent, widowed, divorced, separated, never married), educational attainment (less than high school, regular high school diploma, GED or alternative credential, some college but no degree, associate degree or bachelor’s degree, master’s degree, doctorate degree or professional degree beyond a bachelor’s degree), disability (with a disability, without a disability), employment status (civilian employed at work, civilian employed with a job but not at work, unemployed, armed forces at work, not in labor force, armed forces with a job but not at work); ^b^ Values indicate adjusted odds ratios/relative risks (95% confidence intervals); ^c^ In all logistic regression models, *P* values were < 0.0004 after conservative Bonferroni correction; ^d^ We categorized state as unexpanded states vs. expanded states based on the states’ decisions about adopting the Medicaid expansion as of January 1, 2019; ^e^ Non-US citizens were defined as a citizen of the US or women born abroad of American parents and US-citizens were defined as US citizen by naturalization or women born in the US, Puerto Rico, Guam, the US Virgin Islands, or the Northern Marianas

**Supplementary Table **1 were shown with the results of gaps in Medicaid or any kind of government medical assistance by state among poverty group. Of 19 Medicaid-unexpanded states, 14 (as of 1 January 2019) showed greater-than-average coverage gaps. On the other hand, of a total of 32 expanded states, 3 showed greater-than-average gaps in Medicaid or any kind of government medical assistance.

We also characterize associations between private health coverage and poverty/government financial support in **Supplementary Table **2. In contrast to postpartum women with public health coverage, those with private health insurance or insurance through an employer/union were significantly less likely to live in poverty than other women (aORs, 95% CIs: 0.21, 0.19 ~ 0.23; 0.21, 0.19 ~ 0.23). Also, women with private health insurance or insurance through an employer/union showed significantly lower odds of having government financial support compared to women without those types of insurance. In **Supplementary Tables **3, for the relationships between health insurance coverage and continuous endpoints of poverty level, all the relationship patterns were consistent with the associations between health insurance coverage and poverty status from the logistic regression models.

## Discussion

Our results based on 2019 national representative survey data showed a high prevalence of poverty among postpartum women in the US. The evident positive association (from logistic regression) and positive relationship (from linear regression) between Medicaid or any kind of government medical assistance and poverty showed that on the average, health coverage policies were well implemented to some degree. Our data also showed that many US postpartum women in poverty still did not have Medicaid or any kind of government medical assistance and government financial support. The positive associations (from logistic regression) between each socially vulnerable factor/Medicaid-unexpanded state and no receipt of Medicaid or any kind of government medical assistance indicated that such public health coverage gaps were more likely among postpartum women in poverty who are Asian or some other race (other than White, Black/African American, American Indian/Alaska Native/Native Hawaiian/Other Pacific Islander, or mixed races), who were not US citizens, spoke some language other than English at home, or lived in states that did not adopt Medicaid expansion.

The poverty rate among postpartum women in our study was approximately 10% higher than in the overall population, according to annual poverty reports by the US Census Bureau in which the poverty rate was calculated as we did, in 2019 [22]. Furthermore, the official US poverty rate in 2020 increased up 1.0% point beyond the rate in 2019 [29], which might be attributable to the coronavirus pandemic [30]. Such increases in the number of the poor among the overall population might also imply an increase in the number of postpartum women in poverty. In fact, the recent scoping review found that mothers with children were more likely to suffer from job and financial insecurity compared to both men and women without children during the pandemic [31]. Single mothers in particular suffered from food insecurity more than others during this period [31]. The pandemic-induced recession led to obvious financial hardship in many households [29, 30]. Offering timely postpartum health services to mothers was critical for their overall health [32]. Our findings from examining the implementation status of health insurance coverage are valuable in terms of identifying unmet needs and actual gaps in health and domestic income among postpartum women in poverty.

As expected, Medicaid or any kind of government medical assistance were positively associated with poverty. Since the Affordable Care Act (ACA) expanded Medicaid to cover all adults with income below 138% of the FPL, and we defined poverty among postpartum women as being below 100% of FPL in our study, the evident positive correlations found in our study could be interpreted as natural results and supportive evidence that on the average, health coverage policies were well implemented to some degree. We also observed strong positive associations of Medicaid or any kind of government medical assistance with government financial support. Notably, the magnitude of estimate for the association of Medicaid or any kind of government medical assistance with public assistance income was more than 7 times higher than that with poverty status. This might indicate that the recipients of public assistance were far more likely to be in poverty that included financial hardship related to not just health care costs but also social determinants of health, such as housing and food insecurity [33, 34]. However, although cash public assistance might play a major role in bridging the gaps in the social determinants of health aside from health care access among poor postpartum women, our data showed that a very low proportion of them received public assistance income. In another study, postpartum women insured by Medicaid reported general financial stress, such as concerns about paying monthly utility bills and housing expenses, and financial hardship leading to unmet needs for medical indirect costs and living expenses (e.g., paid parental leave and paid time off work due to medical encounters), although they were largely shielded from out-of-pocket costs for health care [9]. Accordingly, comprehensive public assistance programs should be made more available to postpartum women in poverty, thereby improving the economic resilience of needy households and alleviating adverse birth outcomes [33, 35].

We found that around 35% of postpartum women in poverty were uninsured, with Medicaid or any kind of government medical assistance. Our data also demonstrated larger gaps in Medicaid-unexpanded states than in unexpanded states. Such high uninsurance rates were also found in other studies during the post-ACA Medicaid expansion period (2015 to 2017) [8, 36]. The noncontinuous postpartum insurance rate was 47.5% in expansion states and 57.5% in non-expansion states among women with low incomes [8]. The rate of health insurance disruption among women was 33.9% from preconception to postpartum [36]. Given the gap in uninsurance rates between expansion and nonexpansion states, the adoption status of Medicaid expansion could be one factor contributing to such coverage gaps. In fact, Medicaid eligibility of states without expanded Medicaid programs was quite limited, offering health coverage only to families with FPL < 41% and even considering childless ineligible for it [37]. Thus, postpartum women with household incomes between 41% and 100% of FPL in unexpanded states were left uninsured without any public subsidies because not just the state would not provide Medicaid but the ACA neither gave financial assistance for other coverage only available to people with household incomes of 100 to 400% of FPL [38]. However, it has been shown that adoption of the Medicaid expansion improved postpartum insurance continuity for poor women [8], and insurance continuity is a prerequisite for continuous access to high-quality health care for poor postpartum women [39]. Also, previous studies reported that Medicaid expansions were associated with improved early prenatal care and reduction in adverse birth outcomes [40, 41], and led to improvement in mental health, and reduction in psychological distress among low-income parents [42, 43]. Another possible contributor to health insurance coverage gaps might be changes in insurance coverage over time, called “churning”, such as Medicaid-uninsured churn or Medicaid-private churn during the postpartum period. Medicaid-private churn describes the temporary loss of health coverage in which Medicaid enrollees disenroll and then enroll in private health coverage within a short period of time. Medicaid-uninsured churn indicates the loss of health coverage after Medicaid enrollees disenroll. One previous study [8] found that almost 50% of the decrease in Medicaid-uninsured churn was counterbalanced by Medicaid-private churn in states that expanded Medicaid. Such churning was known to bring about adverse consequences because of varying insurance benefits and provider networks across coverage types [44] and changing the actual provider [45]. Overall, further improved policies targeting potential contributing factors, such as Medicaid expansions and postpartum insurance churn, would be needed to effectively close those coverage gaps and thus improve maternal and infant health across the country.

Our data also showed that among postpartum women in poverty, compared to their White counterparts, women of Asian or some other race had higher rates of public health uninsurance, and women who spoke a language other than English at home showed higher uninsurance rates than those who spoke only English at home. Similarly, another study also reported that Hispanic, Spanish-speaking women comprised almost two-thirds of continuously uninsured women, implying enrollment hurdles related to legal immigration status [46]. However, as the pandemic worsened disproportionate race-related health risks during pregnancy [47], health insurance models should be developed to close the health gaps between races. Our study also showed that postpartum women in poverty without US citizenship had significantly higher rates of public health uninsurance compared with other groups who were citizens. In fact, in 2019, noncitizens, including lawfully present and undocumented immigrants, were significantly more likely to be uninsured than citizens [48]. Moreover, the pandemic likely contributed to a decrease in Medicaid or any kind of government medical assistance among immigrant families [49]. In this regard, improved strategies regarding insurance eligibility for non-US citizens might be needed to minimize the barriers in this group.

Our study has several strengths. First, our data came from a nationwide survey based on a high-quality complex sampling design that controlled coverage, measurement, and processing errors, and we further performed user verification processes to accurately analyze the complex data, ensuring the accuracy and quality of our findings. Second, this survey collected multiple variables related to poverty status, which enabled us to conduct more reliable analyses by adjusting for potential confounding factors. Third, our study investigated state-by-state coverage gaps matching their Medicaid expansion status and examined the association between unexpanded status and lack of receipt of Medicaid or any kind of government medical assistance using all state data. This was the first nationwide investigation on the association, since most previous studies collected data from only several states and explored the impact of Medicaid expansion on health care access [50–52]. We also acknowledged several limitations. First, due to lack of relevant data, we were not able to examine the direct impact of uninsurance on maternal and infant health outcomes or the causes of uninsurance. However, we confirmed coverage gaps and correlation patterns in the US data, which may generate hypotheses for future studies. Second, we were not able to explore patterns of health insurance coverage churn due to the cross-sectional study design. However, we did identify the coverage status at one time point, which will provide evidence for future longitudinal study to better address this topic. Third, because of the lack of information on specific dates of birth and receipts of insurance/coverage plans and longitudinal coverage information, we could not determine how many prepartum women were not covered by Medicaid despite their eligibility during the prenatal or labor and delivery period, which warrants further investigation on such specific coverage gaps in future studies. Lastly, the ACS sample was selected from all counties and county-equivalents in the 50 states using a validated two-phase, two-stage sample design. However, due to the nature of survey data and the sampling process, we could not completely avoid some errors, such as sampling and non-sampling errors, which may introduce bias.

## Conclusions

In summary, our results showed that the receipt of Medicaid or any kind of government medical assistance is significantly associated with poverty and having government financial support. However, there were still large gaps in Medicaid or any kind of government medical assistance among postpartum women in poverty, which indicates that despite improvements, current health insurance–related policies did not yet fully address issues affecting postpartum women who live in poverty. Specifically, among postpartum women in poverty, those living in unexpanded states, those of Asian or some other race (other than White, Black/African American, American Indian/Alaska Native/Native Hawaiian/Other Pacific Islander, or mixed races), non-US citizens, or those who spoke a language other than English at home were likely to have greater gaps in Medicaid or any kind of government medical assistance.

### Electronic supplementary material

Below is the link to the electronic supplementary material.

Supplementary Material 1

## Data Availability

The datasets we used and analyzed in the current study are publicly available on the ACS PUMS website: https://www2.census.gov/programs-surveys/acs/data/pums/2019/1-Year/ .

## Abbreviations

ACA: Affordable Care Act
ACS: American Community Survey
a*β*: Adjusted Coefficient
aOR: Adjusted Odds Ratio
CI: Confidence Interval
FPL: Federal Poverty Level
OECD: Organisation for Economic Co-operation and Development
PUMS: Public Use Microdata Sample
SE: Standard Error
STROBE: Strengthening the Reporting of Observational Studies in Epidemiology
US: United States
