# Supplementary material for: Health insurance coverage and poverty status of postpartum women in the United States in 2019: an ACS-PUMS population-based cross-sectional study
Source: BMC Public Health. 2023 Nov 8;23:2200. doi: 10.1186/s12889-023-17087-4 (PMC10634014; doi:10.1186/s12889-023-17087-4)
Supplement: Supplementary file 1 — Supplementary Material 1 [file 12889_2023_17087_MOESM1_ESM.docx]

**Supplementary Material**

**Health insurance coverage and poverty status of postpartum women in the United States in 2019: An ACS-PUMS population-based cross-sectional study**

Bojung Seo, Jack Edward Turman Jr., Hongmei Nan

**1. Survey Questions for Insurances and Incomes^1^:**

1. **Health Insurance Coverage**

Is this person CURRENTLY covered by any of the following types of health insurance or health coverage plans? Mark "Yes" or "No" for EACH type of coverage in items a – h.

1. Insurance through a current or former employer or union (of this person or another family member): Yes or No
2. Insurance purchased directly from an insurance company (by this person or another family member): Yes or No
3. Medicare, for people 65 and older, or people with certain disabilities: Yes or No
4. Medicaid, Medical Assistance, or any kind of government-assistance plan for those with low incomes or a disability: Yes or No
5. TRICARE or other military health care: Yes or No
6. VA (enrolled for VA health care): Yes or No
7. Indian Health Service: Yes or No
8. Any other type of health insurance or health coverage plan: Yes or No

– Specify (open-ended)

1. **Incomes in the past 12 months**

Mark (X) the "Yes" box for each type of income this person received, and give your best estimate of the TOTAL AMOUNT during the PAST 12 MONTHS. (NOTE: The "past 12 months" is the period from today’s date one year ago up through today.) Mark (X) the "No" box to show types of income NOT received. If net income was a loss, mark the "Loss" box to the right of the dollar amount. For income received jointly, report the appropriate share for each person – or, if that’s not possible, report the whole amount for only one person and mark the "No" box for the other person.

1. Wages, salary, commissions, bonuses, or tips from all jobs. Report amount before deductions for taxes, bonds, dues, or other items: Yes or No

– Specify total amount for past 12 months ($ )

1. Self-employment income from own nonfarm businesses or farm businesses, including proprietorships and partnerships. Report NET income after business expenses: Yes or No

– Specify total amount for past 12 months ($ )

Was that loss?: Yes or No

1. Interest, dividends, net rental income, royalty income, or income from estates and trusts. Report even small amounts credited to an account: Yes or No

– Specify total amount for past 12 months ($ )

Was that loss?: Yes or No

1. Social Security or Railroad Retirement: Yes or No

– Specify total amount for past 12 months ($ )

1. Supplemental Security Income (SSI): Yes or No

– Specify total amount for past 12 months ($ )

1. Any public assistance or welfare payments from the state or local welfare office: Yes or No

– Specify total amount for past 12 months ($ )

1. Retirement income, pensions, survivor or disability income. Include income from a previous employer or union, or any regular withdrawals or distributions from IRA, Roth IRA, 401(k), 403(b), or other accounts specifically designed for retirement. Do not include Social Security: Yes or No

– Specify total amount for past 12 months ($ )

1. Any other sources of income received regularly such as Veterans’ (VA) payments, unemployment compensation, child support or alimony. Do NOT include lump sum payments such as money from an inheritance or the sale of a home: Yes or No

– Specify total amount for past 12 months ($ )

1. **Person’s total incomes in the past 12 months**

What was this person’s total income during the PAST 12 MONTHS? Add entries in questions 2a to 2h; subtract any losses. If net income was a loss, enter the amount and mark (X) the "Loss" box next to the dollar amount:

Is that None? Otherwise specify total amount for past 12 months ($ )

Was that loss?: Yes or No

**Note:** Overall, total personal income examined in this study was a sum added altogether with total amounts of wages, salary, commissions, bonuses, or tips from all jobs before deductions for taxes, bonds, dues, or other items, total self-employment net income from own nonfarm businesses or farm businesses after business expenses, total amounts of interest, dividends, net rental income, royalty income, or income from estates and trusts that were credited to an account, total amounts of social security or railroad retirement, total amounts of supplemental security income, total amounts of any public assistance or welfare payments from the state or local welfare office, total amounts of retirement income, pensions, survivor or disability income including incomes from a previous employer or union, or any regular withdrawals or distributions from IRA, Roth IRA, 401(k), 403(b), or other accounts specifically designed for retirement but not including social security, and total amounts of any other sources of income received regularly such as Veterans’ payments, unemployment compensation, child support or alimony, not including lump sum payments such as money from an inheritance or the sale of a home; any losses were subtracted from the total person’s incomes thereafter.

**2. Estimates of Poverty^2^:**

**How Poverty Is Calculated?**

Poverty status was defined by income-to-poverty ratio determined by using income cutoffs that vary by family size and composition and additionally vary by age in the case of females living alone or with nonrelatives. If a postpartum female’s total family income was less than the appropriate threshold, then the female was considered to be in poverty; similarly, if an unrelated female’s total income was less than the appropriate threshold, then the female was considered to be in poverty. The appropriate poverty thresholds were determined by multiplying the base-year poverty thresholds (1982) by the average of the monthly inflation factors for the 12 months preceding the data collection.

Following the Office of Management and Budget’s (OMB) Statistical Policy Directive 14, the U.S. Census Bureau uses a set of dollar value thresholds that vary by family size and composition to determine who is in poverty.

**Poverty Thresholds for 2019 by Size of Family and Number of Related Children Under 18 Years (in dollars):**

|  | | | | | | | | | |
| --- | --- | --- | --- | --- | --- | --- | --- | --- | --- |
| **Size of family unit** | **Related children under 18 years** | | | | | | | | |
|  | **None** | **One** | **Two** | **Three** | **Four** | **Five** | **Six** | **Seven** | **Eight or more** |
| One person (unrelated individual): |  |  |  |  |  |  |  |  |  |
| Under age 65 | 13,300 |  |  |  |  |  |  |  |  |
| Aged 65 and older | 12,261 |  |  |  |  |  |  |  |  |
| Two people: |  |  |  |  |  |  |  |  |  |
| Householder under age 65 | 17,120 | 17,622 |  |  |  |  |  |  |  |
| Householder aged 65 and older | 15,453 | 17,555 |  |  |  |  |  |  |  |
| Three people | 19,998 | 20,578 | 20,598 |  |  |  |  |  |  |
| Four people | 26,370 | 26,801 | 25,926 | 26,017 |  |  |  |  |  |
| Five people | 31,800 | 32,263 | 31,275 | 30,510 | 30,044 |  |  |  |  |
| Six people | 36,576 | 36,721 | 35,965 | 35,239 | 34,161 | 33,522 |  |  |  |
| Seven people | 42,085 | 42,348 | 41,442 | 40,811 | 39,635 | 38,262 | 36,757 |  |  |
| Eight people | 47,069 | 47,485 | 46,630 | 45,881 | 44,818 | 43,470 | 42,066 | 41,709 |  |
| Nine people or more | 56,621 | 56,895 | 56,139 | 55,503 | 54,460 | 53,025 | 51,727 | 51,406 | 49,426 |
| Source: U.S. Census Bureau. | | | | | | | | | |

If a family’s total money income is less than the applicable threshold, then that family and every individual in it are considered to be in poverty. The official poverty thresholds are updated annually for inflation using the Consumer Price Index for All Urban Consumers (CPI-U). The official poverty definition uses money income before taxes or tax credits and excludes capital gains and noncash benefits (such as Supplemental Nutrition Assistance Program benefits and housing assistance). The thresholds do not vary geographically. *Example:* Suppose Family A comprises five people: two children, their mother, their father, and their great-aunt. Family A’s poverty threshold in 2019 is $31,275. Each member of Family A had the following income in 2019: Mother $11,000 Father $11,000 Great-aunt $10,000 First child 0 Second child 0 Total: $32,000

Since their total family income ($32,000) was higher than their threshold ($31,275), Family A would not be considered “in poverty.”

While the thresholds, in some sense, represent the needs of families, they should be interpreted as a statistical yardstick rather than as a complete description of what people and families need to live. Many government assistance programs use different income eligibility cutoffs. While official poverty rates and the number of people or families in poverty are important, other poverty indicators are considered in the section “Depth of Poverty Measures,” and another approach to setting thresholds and defining resources is discussed in the section “Supplemental Poverty Measure.”

For a history of the official poverty measure, see “Poverty: The History of the Official Poverty Measure” available at <www.census.gov/topics/income-poverty/poverty/about/history-of-the-poverty-measure.html> or “The Development of the Orshansky Poverty Thresholds and Their Subsequent History as the Official U.S. Poverty Measure” by Gordon M. Fisher, available at <www.census.gov/library/working-papers/1997/demo/fisher-02.html>.

**Weighted Average Thresholds:**

Since some data users want a summary of the 48 thresholds to get a general sense of the “poverty line,” the following table provides the weighted average thresholds for 2019. The weighted average thresholds are based on the relative number of unrelated individuals and primary families of each size and composition and are not used in computing poverty estimates (a primary family is a group of two or more people, one of whom is the householder, related by birth, marriage, or adoption and residing together. All such people including related subfamily members are considered as members of one family).

**Weighted Average Poverty Thresholds in 2019:**

| **Size of family unit** | **Dollars** |
| --- | --- |
| One person | 13,011 |
| Two people | 16,521 |
| Three people | 20,335 |
| Four people | 26,172 |
| Five people | 31,021 |
| Six people | 35,129 |
| Seven people | 40,016 |
| Eight people | 44,461 |
| Nine people or more | 52,875 |

Source: U.S. Census Bureau.

**3. The detailed list of Medicaid expansion states:**

The expanded states were Alaska, Arizona, Arkansas, California, Colorado, Connecticut, Delaware, District of Columbia, Hawaii, Illinois, Indiana, Iowa, Kentucky, Louisiana, Maryland, Massachusetts, Michigan, Minnesota, Montana, Nevada, New Hampshire, New Jersey, New Mexico, New York, North Dakota, Ohio, Oregon, Pennsylvania, Rhode Island, Vermont, Washington, and West Virginia. Unexpanded states were Alabama, Florida, Georgia, Idaho, Kansas, Maine, Mississippi, Missouri, Nebraska, North Carolina, Oklahoma, South Carolina, South Dakota, Tennessee, Texas, Utah, Virginia, Wisconsin, and Wyoming.

**Supplementary Table 1. Medicaid or any kind of government-medical assistance gaps in US postpartum women in poverty by state**

| **States** | **No Medicaid ^a^/Total** | | **% ^e^** | **Status of Medicaid Expansion Decision^3 b^** |
| --- | --- | --- | --- | --- |
|  | **N ^c^** | **SE ^d^** |  |  |
| **Alabama/AL** | 6343/13190 | 1168/1491 | 48.1 | Not Adopted |
| **Alaska/AK** | 428/1776 | 218/642 | 24.1 | Adopted |
| **Arizona/AZ** | 7350/20243 | 1223/2063 | 36.3 | Adopted |
| **Arkansas/AR** | 4310/12522 | 1030/1579 | 34.4 | Adopted |
| **California/CA** | 19907/74845 | 1791/3595 | 26.6 | Adopted |
| **Colorado/CO** | 4173/10255 | 1121/1603 | 40.7 | Adopted |
| **Connecticut/CT** | 1713/5437 | 707/1119 | 31.5 | Adopted |
| **Delaware/DE** | 628/2117 | 381/666 | 29.7 | Adopted |
| **District of Columbia/DC** | 67/1451 | 69/530 | 4.6 | Adopted |
| **Florida/FL** | 20414/44272 | 2059/3098 | 46.1 | Not Adopted |
| **Georgia/GA** | 14720/28663 | 1410/1907 | 51.4 | Not Adopted |
| **Hawaii/HI** | 681/2344 | 304/623 | 29.1 | Adopted |
| **Idaho/ID** | 4113/5914 | 856/1076 | 69.5 | Not Adopted |
| **Illinois/IL** | 7430/26999 | 1525/2750 | 27.5 | Adopted |
| **Indiana/IN** | 5139/16053 | 943/1516 | 32 | Adopted |
| **Iowa/IA** | 2455/7311 | 697/1146 | 33.6 | Adopted |
| **Kansas/KS** | 4179/8576 | 812/1248 | 48.7 | Not Adopted |
| **Kentucky/KY** | 1939/13762 | 512/1639 | 14.1 | Adopted |
| **Louisiana/LA** | 5146/18199 | 977/2212 | 28.3 | Adopted |
| **Maine/ME** | 478/2610 | 259/667 | 18.3 | Not Adopted |
| **Maryland/MD** | 2829/9993 | 821/1795 | 28.3 | Adopted |
| **Massachusetts/MA** | 957/8308 | 401/1073 | 11.5 | Adopted |
| **Michigan/MI** | 5480/27710 | 1095/2658 | 19.8 | Adopted |
| **Minnesota/MN** | 1260/7587 | 436/1394 | 16.6 | Adopted |
| **Mississippi/MS** | 4079/12987 | 744/1557 | 31.4 | Not Adopted |
| **Missouri/MO** | 6888/17593 | 1063/2022 | 39.2 | Not Adopted |
| **Montana/MT** | 510/2525 | 224/743 | 20.2 | Adopted |
| **Nebraska/NE** | 1476/4481 | 591/902 | 32.9 | Not Adopted |
| **Nevada/NV** | 3371/8444 | 823/1192 | 39.9 | Adopted |
| **New Hampshire/NH** | 211/717 | 147/307 | 29.4 | Adopted |
| **New Jersey/NJ** | 5789/16333 | 1153/1622 | 35.4 | Adopted |
| **New Mexico/NM** | 3093/8454 | 932/1210 | 36.6 | Adopted |
| **New York/NY** | 8132/43874 | 1221/2817 | 18.5 | Adopted |
| **North Carolina/NC** | 14733/35979 | 1492/2624 | 40.9 | Not Adopted |
| **North Dakota/ND** | 255/3263 | 200/981 | 7.8 | Adopted |
| **Ohio/OH** | 6820/36357 | 1114/3026 | 18.8 | Adopted |
| **Oklahoma/OK** | 8850/15045 | 1470/1783 | 58.8 | Not Adopted |
| **Oregon/OR** | 2445/7372 | 709/1244 | 33.2 | Adopted |
| **Pennsylvania/PA** | 9004/35457 | 1450/3053 | 25.4 | Adopted |
| **Rhode Island/RI** | 390/486 | 237/244 | 80.2 | Adopted |
| **South Carolina/SC** | 6793/16004 | 1294/2185 | 42.4 | Not Adopted |
| **South Dakota/SD** | 1181/2309 | 466/596 | 51.1 | Not Adopted |
| **Tennessee/TN** | 5502/19798 | 1010/1715 | 27.8 | Not Adopted |
| **Texas/TX** | 56858/87466 | 3255/4260 | 65.0 | Not Adopted |
| **Utah/UT** | 2156/5321 | 556/882 | 40.5 | Not Adopted |
| **Vermont/VT** | 240/1222 | 183/497 | 19.6 | Adopted |
| **Virginia/VA** | 7690/17040 | 1090/1527 | 45.1 | Not Adopted |
| **Washington/WA** | 3641/16815 | 769/1579 | 21.7 | Adopted |
| **West Virginia/WV** | 849/5412 | 314/960 | 15.7 | Adopted |
| **Wisconsin/WI** | 2609/10689 | 770/1756 | 24.4 | Not Adopted |
| **Wyoming/WY** | 365/1014 | 181/252 | 36.0 | Not Adopted |
| **Total** | 286069/802594 | 8273/13560 | 35.6 |  |

Abbreviation: N, number; SE, standard errors

Note: Poverty (<100%) vs no poverty (≥100%) was defined according to income-to-poverty ratio.

^a^ Medicaid means Medicaid, medical assistance, or any kind of government-assistance for those with low incomes or a disability; ^b^ States' decisions about adopting the Medicaid expansion are as of January 1, 2019; ^c^ Weighted estimates; ^d^ Standard errors of weighted frequency; ^e^ percent of postpartum women in poverty with no Medicaid, medical assistance, or any kind of government-assistance for those with low incomes or a disability.

**Supplementary Table 2. Associations between private health insurance and poverty/government financial support** ^d^

|  | **Poverty** ^a^ | | **Having public assistance income** ^b^ | | **Having supplementary security income** ^b^ | |
| --- | --- | --- | --- | --- | --- | --- |
|  | **No** | **Yes** | **No** | **Yes** | **No** | **Yes** |
| **Private health insurance (Ref.: no)** |  |  |  |  |  |  |
| Yes, n (%) | 2258606 (74.4) | 147239 (18.3) | 2375878 (98.8) | 29967 (1.2) | 2393822 (99.5) | 12023 (0.5) |
| No, n (%) | 778070 (25.6) | 655355 (81.7) | 1317675 (91.9) | 115750 (8.1) | 1397029 (97.5) | 36396 (2.5) |
| Age and race adjusted model ^c^ | Ref | 0.09 (0.08~0.09) | Ref | 0.16 (0.13~0.19) | Ref | 0.18 (0.14~0.23) |
| Multivariable adjusted model ^c^ | Ref | 0.21 (0.19~0.23) | Ref | 0.28 (0.22~0.35) | Ref | 0.58 (0.40~0.84) |
| **Insurance through a current or former employer or union (Ref.: no)** |  |  |  |  |  |  |
| Yes, n (%) | 2012781 (66.3) | 109045 (13.6) | 2096940 (98.8) | 24886 (1.2) | 2112442 (99.6) | 9384 (0.4) |
| No, n (%) | 1023895 (33.7) | 693549 (86.4) | 1596613 (93.0) | 120831 (7.0) | 1678409 (97.7) | 39035 (2.3) |
| Age and race adjusted model ^c^ | Ref | 0.09 (0.08~0.10) | Ref | 0.18 (0.15~0.22) | Ref | 0.18 (0.14~0.23) |
| Multivariable adjusted model ^c^ | Ref | 0.21 (0.19~0.23) | Ref | 0.30 (0.24~0.39) | Ref | 0.50 (0.36~0.71)^e^ |

Note: Poverty (<100%) vs no poverty (≥100%) was defined according to income-to-poverty ratio.

^a^ In multivariable adjusted logistic models for poverty status, we adjusted age (continuous), race (white alone, black or African American alone , others), region (northeast, midwest, south, west), nativity (native, foreign born), marital status (now married spouse present, now married spouse absent, widowed, divorced, separated, never married), educational attainment (less than high school, regular high school diploma, GED or alternative credential, some college but no degree, associate degree or bachelor degree, master degree, doctorate degree or professional degree beyond a bachelor degree), language other than English spoken at home (yes speaks another language, no speaks only English), ambulatory difficulty (yes, no), cognitive difficulty (yes, no), disability (with a disability, without a disability), employment status (civilian employed at work, civilian employed with a job but not at work, unemployed, armed forces at work, not in labor force, armed forces with a job but not at work); ^b^ In multivariable adjusted logistic models for having public assistance income or having supplementary security income, we excluded region and employment status and added class of worker (employee of a private for-profit company or business, or of an individual, for wages, salary, or commissions, employee of a private not-for-profit, tax-exempt, or charitable organization, local government employee, state government employee, federal government employee, self-employed in own not incorporated business professional practice or farm, self-employed in own incorporated business professional practice or farm, working without pay in family business or farm, unemployed and last worked 5 years ago or earlier or never worked) for adjustment; ^c^ Values indicate adjusted odds ratios (95% confidence intervals); ^d^ In all models, *P* values were <0.0002 after conservative Bonferroni correction; ^e^ As an exception, *P* value=0.0002 after the conservative Bonferroni correction.

**Supplementary Table 3. Relationships between health insurance coverage and total person's income/income-to-poverty ratio** ^c^

|  | **Total person's income** | **Income-to-poverty ratio** |
| --- | --- | --- |
| **Public health coverage, yes vs. no** |  |  |
| Weighed means (Yes/No) | 11763/37391 | 141/328 |
| Age and race adjusted model ^b^ | -20352 (-21225 ~ -19478) | -164 (-168 ~ -159) |
| Multivariable adjusted model ^b^ | -5366 (-6162 ~ -4569) | -87 (-92 ~ -82) |
| **Medicaid, Medical Assistance, or any kind of government-assistance, yes vs. no** |  |  |
| Weighed means (Yes/No) | 11437/37260 | 138/327 |
| Age and race adjusted model ^b^ | -20411 (-21295 ~ -19527) | -165 (-170 ~ -161) |
| Multivariable adjusted model ^b^ | -5446 (-6254 ~ -4638) | -88 (-93 ~ -83) |
| **Private health insurance, yes vs. no** |  |  |
| Weighed means (Yes/No) | 41126/11081 | 350/143 |
| Age and race adjusted model ^b^ | 25758 (24851 ~ 26666) | 189 (185 ~ 193) |
| Multivariable adjusted model ^b^ | 6831 (5997 ~ 7665) | 105 (100 ~ 110) |
| **Insurance through a current or former employer or union, yes vs. no** |  |  |
| Weighed means (Yes/No) | 43485/13135 | 360/166 |
| Age and race adjusted model ^b^ | 26396 (25474 ~ 27318) | 177 (173 ~ 181) |
| Multivariable adjusted model ^b^ | 8127 (7295 ~ 8959) | 97 (93 ~ 101) |

Note: Poverty (<100%) vs no poverty (≥100%) was defined according to income-to-poverty ratio.

^a^ In multivariable adjusted linear models, we adjusted age (continuous), race (white alone, black or African American alone , others), region (northeast, midwest, south, west), nativity (native, foreign born), marital status (now married spouse present, now married spouse absent, widowed, divorced, separated, never married), educational attainment (less than high school, regular high school diploma, GED or alternative credential, some college but no degree, associate degree or bachelor degree, master degree, doctorate degree or professional degree beyond a bachelor degree), language other than English spoken at home (yes speaks another language, no speaks only English), ambulatory difficulty (yes, no), cognitive difficulty (yes, no), disability (with a disability, without a disability), employment status (civilian employed at work, civilian employed with a job but not at work, unemployed, armed forces at work, not in labor force, armed forces with a job but not at work). ^b^ Values indicate adjusted β (95% Confidence Intervals); ^c^ All *P* values were <0.0004 after conservative Bonferroni correction.

**References**

1. ACS Questionnaire 2019. Census.gov; <https://www2.census.gov/programs-surveys/acs/methodology/questionnaires/2019/quest19.pdf>

2. Jessica Semega MK, Emily A. Shrider, and John F. Creamer. *Income and Poverty in the United States: 2019* 2020. *Current Population Reports*. September 15, 2020. <https://www.census.gov/content/dam/Census/library/publications/2020/demo/p60-270.pdf>

3. Status of State Action on the Medicaid Expansion Decision. Kaiser Family Foundation. Updated July 21, 2022. Accessed August 2, 2022, 2022. <https://www.kff.org/health-reform/state-indicator/state-activity-around-expanding-medicaid-under-the-affordable-care-act/?currentTimeframe=0&sortModel=%7B%22colId%22:%22Location%22,%22sort%22:%22asc%22%7D>
